# A Resource for the Transcriptional Signature of Bona Fide Trophoblast Stem Cells and Analysis of Their Embryonic Persistence

**DOI:** 10.1155/2015/218518

**Published:** 2015-12-14

**Authors:** Georg Kuales, Matthias Weiss, Oliver Sedelmeier, Dietmar Pfeifer, Sebastian J. Arnold

**Affiliations:** ^1^Renal Department, Centre for Clinical Research, University Medical Centre, Breisacher Strasse 66, 79106 Freiburg, Germany; ^2^Department of Hematology, Oncology and Stem Cell Transplantation, University Medical Centre, Freiburg, Germany; ^3^Institute of Experimental and Clinical Pharmacology and Toxicology, University of Freiburg, Freiburg, Germany; ^4^BIOSS, Centre for Biological Signalling Studies, Albert Ludwigs University, Freiburg, Germany

## Abstract

Trophoblast stem cells (TSCs) represent the multipotent progenitors that give rise to the different cells of the embryonic portion of the placenta. Here, we analysed the expression of key TSC transcription factors *Cdx2*, *Eomes*, and *Elf5* in the early developing placenta of mouse embryos and in cultured TSCs and reveal surprising heterogeneity in protein levels. We analysed persistence of TSCs in the early placenta and find that TSCs remain in the chorionic hinge until E9.5 and are lost shortly afterwards. To define the transcriptional signature of bona fide TSCs, we used inducible gain- and loss-of-function alleles of *Eomes* or *Cdx2*, and *Eomes* ^GFP^, to manipulate and monitor the core maintenance factors of TSCs, followed by genome-wide expression profiling. Combinatorial analysis of resulting expression profiles allowed for defining novel TSC marker genes that might functionally contribute to the maintenance of the TSC state. Analyses by qRT-PCR and *in situ* hybridisation validated novel TSC- and chorion-specific marker genes, such as *Bok/Mtd, Cldn26, Duox2, Duoxa2, Nr0b1*, and *Sox21*. Thus, these expression data provide a valuable resource for the transcriptional signature of bona fide and early differentiating TSCs and may contribute to an increased understanding of the transcriptional circuitries that maintain and/or establish stemness of TSCs.

## 1. Introduction

Trophectoderm (TE) and inner cell mass (ICM) are the first cell lineages that are specified at the 16- to 32-cell (morula) stage around embryonic day 3 (E3.0) of mouse development (reviewed in [1, 2]). Mutual negative feedback inhibition of the two key transcription factors* Cdx2* and* Oct4* establishes the lineages of* Cdx2*-expressing TE and* Oct4*-positive ICM cells [3–6]. Multiple signals, including cell polarity, cell-cell contacts, positional information, and Hippo signalling, converge on the phosphorylation state of Yes-associated protein (YAP). YAP forms a transcriptional complex with TEAD4 to activate* Cdx2* transcription in predominantly outer, peripheral cells of the early embryo, thus priming the TE programme [4–13]. Additional layers of regulation ensure lineage restricted* Cdx2* and* Oct4* expression, such as Notch-signalling, which cooperates with TEAD4 to directly regulate* Cdx2* expression [14]. Similarly, the transcription factor* AP2γ* promotes* Cdx2* expression and downregulates Hippo signalling [15]. GATA3 acts in parallel with CDX2 and downstream of TEAD4 to induce an overlapping set of target genes in the trophoblast lineage [16]. Within the polar TE, which is defined by its vicinity to the ICM at the blastocyst stage (E3.5), a population of trophoblast stem cells (TSCs) is established and maintained by ICM-derived Fgf- and Nodal-signals, to generate the main cellular source for formation of the embryonic part of the placenta [13, 17–20]. Following initial specification of TE fate by* Cdx2*-functions, additional transcriptional regulators, including* Eomes* [4] and* Elf5* [21], initiate expression in the TE lineage or become specifically restricted to TE cells such as* AP2γ* [22, 23]. Following implantation around E4.5, the polar TE gives rise to the extraembryonic ectoderm (ExE), which contains TSCs, and the ectoplacental cone (EPC), which mediates the embryonic invasion into the decidual wall then connects the embryo to the maternal uterus. TSCs retain their stem cell characteristics, namely, self-renewal capacity and multipotency, only in the polar TE and the ExE while cells of the mural TE lack supportive signalling from the ICM and thus differentiate to primary trophoblast giant cells (TGCs) [17]. TSC maintenance is controlled by transcriptional circuitries involving* Elf5* [24, 25],* Ets2* [26], and* AP2γ* [23, 27], which act in positive feed forward loops to maintain* Cdx2* and/or* Eomes* expression. After E7.5, the core set of TSC marker genes, including* Cdx2*,* Eomes*,* Elf5*, and* Esrrβ*, remain expressed in the chorion in addition to other embryonic and extraembryonic expression domains [24, 28–30].

TSCs can be routinely isolated and cultured from E3.5 blastocysts and E6.5 ExE explants under defined conditions [17]. Assessing the potential to isolate cells with TSC character beyond E6.5, Uy and colleagues have shown that the frequency to obtain TSC clones increases until the 4-somite pair stage (~E8.0 to E8.25) but then rapidly drops and no TSCs can be isolated following the 11-somite pair stage around E8.5 to E8.75 [31]. The T-box transcription factor* Eomes* was previously used to mark TSCs through different developmental stages and expression of an* Eomes*::*GFP* BAC transgene was detected until E14.5 in the outer periphery of the murine placenta [32]. To date it was not clearly shown until which embryonic stage cells with TSC character are maintained in the TSC niche, which is functionally defined by high levels of Fgf and Tgf*β* signals [17, 33]. In addition to* Eomes* [34] and* Cdx2* [35], also other transcription factors share essential functions for TSC self-renewal and multipotency including* Elf5* [24],* Esrrβ* [29],* Ets2* [26],* AP2γ* [23, 27, 36], and* Sox2* [37]. A recent report demonstrated that the functional loss of the histone demethylase* Lsd1* in TSCs results in premature migration of TSCs from their niche, demonstrating additional requirements of proper epigenetic regulation for the propagation of TSCs [38, 39].

In addition to their functional importance during TSC maintenance,* Cdx2* [3, 11],* Eomes* [3],* Elf5* [25],* Tead4* [11],* AP2γ* [23], and* Gata3* [16] also evoke lineage conversion from mouse embryonic stem (mES) cells to the TE lineage when overexpressed (reviewed in [40]). While the transcriptional programme involving* Cdx2*,* Eomes*,* Elf5*,* AP2γ*, and* Ets2* is key for the maintenance of the trophoblast stemness state, genetic deletions of each of these transcription factors generate remarkable different embryonic phenotypes.* Cdx2*-deficiency results in implantation defects [41],* Eomes*-deficient embryos show early postimplantation [34], and the deletions of* Elf5* [24],* Ets2* [26, 42, 43], or* AP2γ* [36] lead to postgastrulation lethality. This diversity in loss-of-function phenotypes might indicate differential requirements of these factors for the stemness maintaining regulatory circuitry. Alternatively, different states of TSCs might exist with differential developmental potential and distinct requirements of transcriptional regulation, in analogy to different states of pluripotency, such as that found in naïve or primed pluripotent stem cells [39, 44].

In the current study, we have analysed the endogenous expression of key stemness-maintaining factors,* Cdx2*,* Eomes*, and* Elf5*, in the chorion of gastrulation stage embryos to characterise TSCs by overlapping marker gene expression. We traced* Eomes* expression through later gastrulation stages and demonstrate that it is lost around E9.5 in the region emanating from the chorionic hinge. Surprisingly, TSC markers consistently show heterogenous, partially nonoverlapping expression in different areas of the late TSC niche, potentially indicating different states of TSCs during placental development. The analysis of TSCs cultured under stemness-maintaining conditions additionally revealed heterogeneous TSC marker expression* in vitro*, underscoring our findings from embryonic analyses in the chorion. To determine the transcriptional state that describes undifferentiated, bona fide TSCs and to monitor transcriptional changes during early TSC differentiation, we used genetic tools to manipulate* Eomes* and* Cdx2* expression in TSC cultures followed by genome wide transcriptional profiling. First, we generated TSCs that harbour an *Eomes* ^GFP^ reporter allele, thereby marking* bona fide* TSCs that were enriched by fluorescence activated cell sorting (FACS) and forced towards differentiation by removal of stemness maintaining conditions. Second, we employed TSCs that allow for the inducible deletion of* Eomes* gene function, and third we used inducible expression of key TSC transcriptional regulators* Cdx2* and* Eomes* in mouse ES (mES) cells for the identification of downstream target genes. Resulting differential expression profiles allow for a detailed description of the TSC signature and the changes during early differentiation, which might be directly or indirectly regulated by a combination of Fgf- and Tgf*β*-signalling and the key regulatory factors of TSCs,* Cdx2*, and* Eomes*. We used resulting expression profiles to identify novel candidate TSC marker genes at stemness state. To validate this approach, a handful of differentially expressed genes were selected and tested by qRT-PCRs of bona fide TSCs and during early differentiation. Additionally, expression of novel candidates was analysed in the TSC compartment of postimplantation embryos* by in situ* hybridisation analysis. In summary, this study characterises and describes the expression signature of TSCs within the embryo and in cultured TSCs.

## 2. Materials and Methods

### 2.1. Cell Culture

Genetically modified TSCs were isolated from E3.5 blastocysts of animals carrying alleles for *Eomes* ^GFP^ [45], *Eomes* ^CA^ [47], and R26Cre^ER^ [58] according to standard protocols [17]. TSCs cultured in stemness maintaining conditions (SMC) were kept in 70% mouse embryonic fibroblast- (MEF-) conditioned TSC-medium (MCM) supplemented with hrFGF4 and heparin (F4H) on MEFs [17]. For differentiation conditions (DC), TSCs were cultured in TSC-medium without MCM and F4H on gelatinized plates without feeder cells [17]. Cre-mediated excision of the conditional *Eomes* ^CA^ allele was induced by administration of 1 *μ*g/mL 4-hydroxytamoxifen (Sigma Aldrich, H7904) to the culture medium for up to 3 days.

To generate mES cells that inducibly express* Cdx2* or* Eomes* in response to doxycycline administration, we performed inducible cassette exchange (ICE) using A2lox.cre mES cells [59] and the p2lox-V5 vector system using gateway cloning (Invitrogen) [48]. Expression was induced by administration of 1 *μ*g/mL doxycycline (Fargon, 137087) to the ES cell culture medium composed of DMEM (Gibco, 11960), 15% ES cell-qualified FBS (Gibco, 16141), 2 mM L-glutamine (Gibco, 25030), 50 U/mL penicillin/50 *μ*g/mL streptomycin (Gibco, 15070), 0.1 mM nonessential amino acids (Gibco, 11140), 1 mM sodium pyruvate (Gibco, 11360), 0.1 mM *β*-mercaptoethanol (Sigma Aldrich, M7522), and 1,000 U/mL leukemia inhibitory factor (Merck Millipore, ESG1107).

### 2.2. Flow Cytometry

Single cell suspensions of *Eomes* ^GFP^ TSCs cultured in SMC were FACS-purified for GFP^high^ cells using a MoFlo Legacy cell sorter (Beckman Coulter). Selected cells were reseeded in SMC without MEF feeder cells for 24 h before preparation of the RNA.

### 2.3. Immunofluorescence Staining of E7.5–E14.5 Tissue Sections

Whole decidua (E6.5–E8.5) or placenta (E10.5–E14.5) was isolated from *Eomes* ^GFP^ pregnant females on ice, washed in PBS, and fixed in 4% PFA at 4°C for 1 h (E6.5, E7.5) or 3 h (E8.5–E14.5). Fixed tissues were rinsed in PBS and successively transferred to 15% and 30% sucrose dissolved in PBS until samples no longer floated. Tissues were subsequently incubated in embedding medium (7.5% fish skin gelatin, 15% sucrose) for 1 h at 37°C and snap frozen in liquid nitrogen. Frozen blocks were cut at 8 *μ*m sections on a cryostat (CM3050s, Leica), and sections mounted on SuperFrost Plus slides (R. Langenbrinck, 03-0060). Washing steps, blocking, incubation with primary and secondary antibodies, and DAPI staining were performed as described for immunofluorescence on TSCs.

### 2.4. Immunofluorescence Staining on TSCs

TSC cultures were grown on coverslips coated with 0.1% gelatin, briefly rinsed in PBS, and fixed in 4% PFA for 30 min on ice. Fixed cells were washed with PBS containing 0.1% Tween-20 (PBS-T), blocked for 1 h in PBS-T with 5% bovine serum albumin (BSA-T), and incubated with primary antibodies (Supplementary Table S1 available online at http://dx.doi.org/10.1155/2015/218518) in 5% BSA-T over night at 4°C. After washing off the primary antibodies, samples were incubated for 45 min at RT with conjugated secondary antibodies (Supplementary Table S1) diluted in 5% BSA-T. Cells were counterstained for 10 min at RT with DAPI diluted 1 : 1000 in PBS and mounted with Fluoromount-G. Fluorescent images were captured on a Zeiss Axiovert 200 M, equipped with a 20x Plan-Apochromat objective. Displayed images were eventually merged and brightness adjusted using Adobe Photoshop CS5 software.

### 2.5. RNA Preparation for Microarray and Quantitative RT-PCR

Total RNA was purified from TSC and ES cell culture dishes using the RNeasy Mini Kit (QIAGEN, 74104) using 350 *μ*L RLT lysis buffer per 6 cm culture dish. On-column DNaseI digestion was applied and RNA eluted in 50 *μ*L RNase free water, yielding concentrations of 179–1.686 ng/*μ*L RNA.

### 2.6. Microarray and Analysis of Microarray Data

To identify genes overrepresented in TSCs, gene expression datasets obtained from the microarray were sorted for highest negative (*Eomes* ^GFP^ TSC differentiation and *Eomes* ^CA^) or positive (induced* Eomes* or* Cdx2* expression) fold changes. Resulting lists from induced* Eomes* deletion or induced* Cdx2* or* Eomes* expression were additionally filtered for genes that were regulated at least 1.5-fold when compared to respective controls. Only genes with *p* values ≤ 0.05 were considered for further analyses. Finally, median expression values of mES cell- and TSC-based experimental subsets were calculated for each gene. Gene lists were compared by Venn diagrams using the Manteia data mining system (http://manteia.igbmc.fr/).

### 2.7. Validation by Quantitative RT-PCR and Statistical Analyses

For reverse transcription, the QIAGEN QuantiTect Reverse Transcription Kit (205311) was used according to the protocol with an input of 1.0 *μ*g RNA per sample. Quantitative PCR was carried out in triplicate from three independent experiments, using the Roche Light Cycler 480 (SN: 1126) detection system and the Light Cycler 480 DNA SYBR Green I Master kit (Roche, 04707516001) and the primers listed in Supplementary Table S2.* 36B4* and *βcat* served as reference genes. mRNA expression levels over time were calculated relative to day 0 which was set to 1. Statistical analyses were performed using a two-sided Student's *t*-test and the standard error of the mean was calculated for each dataset consisting of biological triplicates in technical triplicates each, individually.

### 2.8. Validation by* In Situ* Hybridization

Deciduae containing embryos were dissected and fixed in 4% PFAin PBS, dehydrated through ethanol series, and embedded in paraffin before 8 *μ*M sections were prepared on a Leica RM2165 microtome.* In situ* hybridization on paraffin sections was performed according to standard protocols [60] using* Bok/Mtd*-,* Cldn26*-,* Cyp26a1*-,* Duox2*-,* Duoxa2*-,* Eomes*-,* Nr0b1*-, and* Sox21*-specific probes. Eosin counterstaining was performed according to standard protocols [60].

### 2.9. Animals

All mice were housed in the pathogen-free barrier facility of the University Medical Centre Freiburg in accordance with the institutional guidelines and approval by the regional board.

## 3. Results

### 3.1. Bona Fide TSCs Are Marked by Coexpression of* Eomes*,* Cdx2*, and* Elf5* and Are Lost from the Chorionic Hinge around E9.5

**Figure 1:**
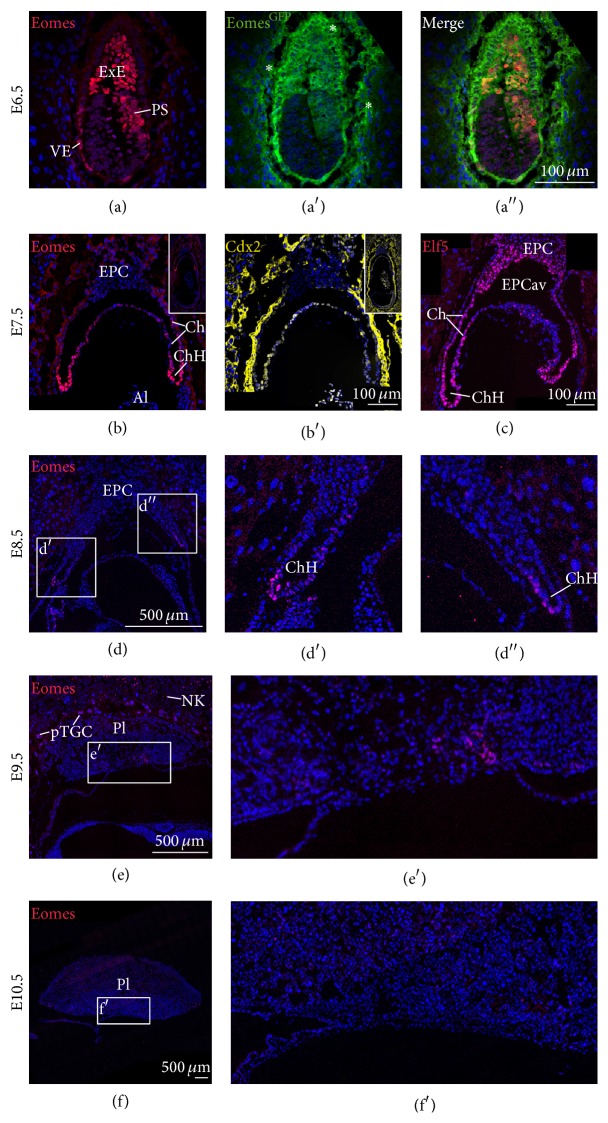
EOMES colocalizes with TSC markers CDX2 and ELF5 in the E7.5 chorionic hinge and expression is lost around E9.5. Immunofluorescence staining shows nuclear EOMES and *Eomes* ^GFP^ reporter expression in (a–a′′) the visceral endoderm (VE), the primitive streak (PS), and the extraembryonic ectoderm (ExE) at E6.5, (b) within the chorion (Ch) and the chorionic hinge at E7.5, and (d–d′′) in the chorion at E8.5. Asterisks in (a′) indicates unspecific signals in extraembryonic tissues. (b and b′) TSC markers EOMES and CDX2 colocalize in cells of the chorion at E7.5. EOMES staining is most prominent in the chorionic hinge and shows a gradient along the chorion, while CDX2 does not show graded reduction along the chorion. (c) ELF5 similarly localizes to the chorion with strongest staining in the chorionic hinge and additionally expands into the ectoplacental cone (EPC) adjacent to the ectoplacental cavity (EPCav). (e, e′) Faint EOMES staining can be detected at E9.5 in the region emanating from the chorionic hinge, and (f, f′) staining is entirely lost at E10.5. (e) EOMES can be additionally found in parietal trophoblast giant cells (pTGC) and natural killer cells (NK) at E9.5. Al, allantois; EPC, ectoplacental cone; EPCav, ectoplacental cavity ExE, extraembryonic ectoderm; Ch, chorion; ChH, chorionic hinge; Pl, placenta; PS, primitive streak; pTGC, parietal trophoblast giant cells VE, visceral endoderm; NK, NK cells.

To investigate the spatial and temporal distribution of TSCs during placentogenesis, we performed detailed immunofluorescence (IF) staining using antibodies specific for EOMES, CDX2, and ELF5, in combination with a previously described *Eomes* ^GFP^ reporter allele [45]. EOMES protein and the GFP-reporter can be detected in the TSC compartment of the extraembryonic ectoderm (ExE), in the primitive streak (PS), and in the visceral endoderm (VE) during early gastrulation at E6.5 (Figures 1(a)–1(a′′)) [45]. One day later, at E7.5* Eomes* expression is refined to a subregion of the chorion, which we refer to as the chorionic hinge (ChH), and expression is gradually reduced in the remainder of the chorion (Figure 1(b)). CDX2 colocalizes with EOMES within the chorion at E7.5 but, unlike EOMES, the staining intensity shows no gradual changes and is uniform throughout the entire chorion (Figure 1(b′)). CDX2 is additionally found in the extraembryonic mesoderm of the allantois (Figure 1(b′)). Finally, we stained for expression of* Elf5*, which acts in the transcriptional circuitry that maintains TSC stemness by positive feed-forward regulation of* Eomes* and* Cdx2* expression [25]. Strongest ELF5 staining was detected in the ChH and the distal portion of the ectoplacental cone (EPC) facing the ectoplacental cavity while reduced levels are found throughout the remainder of the chorion and the proximal EPC (Figure 1(c)). At E8.5, EOMES expressing cells are remaining within the tip of the ChH, which is detached from the surrounding tissues, while the central part of the chorion has started to fuse with the allantois and the EPC (Figures 1(d)–1(d′′)). EOMES-positive cells are almost entirely lost around E9.5 from the forming placenta (Figures 1(e) and 1(e′)) with only little EOMES staining in a region emanating from the former ChH (Figure 1(e′)). Following E10.5 placental EOMES staining could not be detected, apart from single EOMES positive cells that most likely represent placental natural killer cells (Figures 1(e) and 1(f)) [46]. In summary, these detailed marker analyses suggest that EOMES-positive TSCs remain in the chorionic hinge as the functional stem cell niche of TSCs until E9.5 and are not found at later time points.

### 3.2. TSCs in Culture Show Heterogeneous Staining for Stemness Factors and Can Be Identified by High Levels of the *Eomes* ^GFP^ Reporter Expression

**Figure 2:**
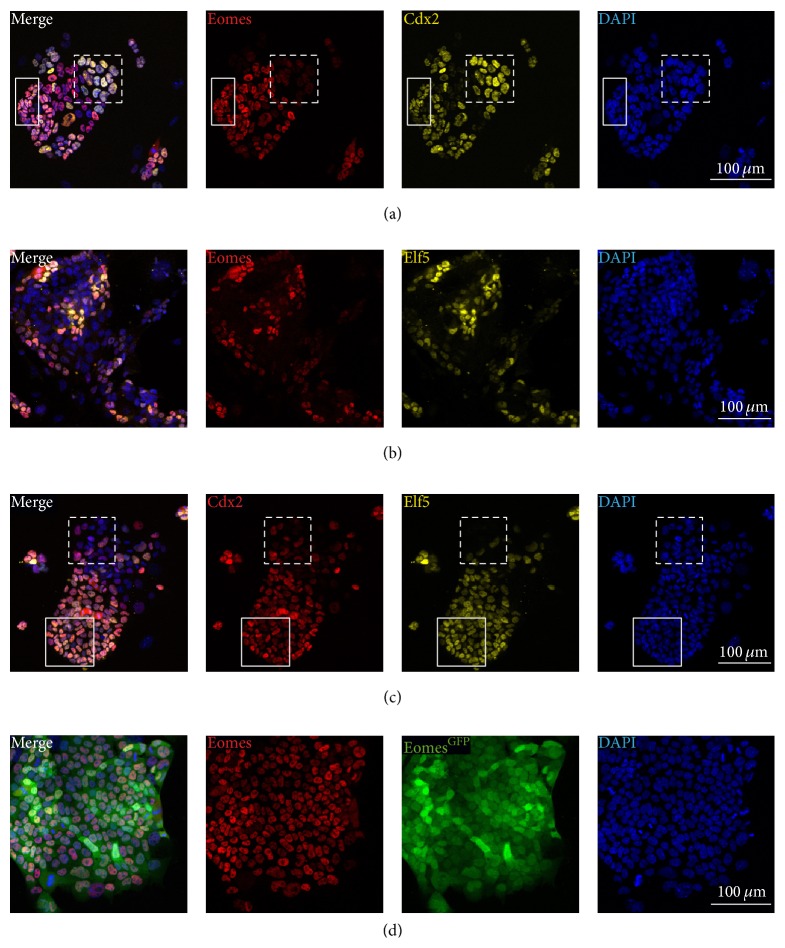
TSCs in culture exhibit heterogeneous patterns of EOMES, CDX2, and ELF5 staining which is recapitulated by *Eomes* ^GFP^ reporter expression. Cultured TSCs are colabelled with antibodies against EOMES, CDX2, and ELF5 or the *Eomes* ^GFP^ knock-in reporter allele. (a) EOMES staining and CDX2 staining largely overlap in TSCs of small size, which morphologically match undifferentiated TSCs (solid boxed region), but staining diverges in enlarged cells (dashed boxed region). (b) ELF5 and EOMES immunostaining widely labels the same cells, and accordingly (c) ELF5 and CDX2 do not colocalize in enlarged cells ((c), dashed boxed region). (d) EOMES protein levels and *Eomes* ^GFP^ reporter expression closely correlate and highest fluorescence intensities are found in cells with strongest EOMES staining.

TSCs can be cultured under conditions of continuous stimulation with Fgf- and Tgf*β*- growth factors for extended periods of time and multiple passages [17, 33]. However, TSCs* in vitro* exhibit an eminent tendency towards spontaneous differentiation and in stemness-maintaining conditions about 5–10% of TSCs differentiate towards the trophoblast giant cell fate, indicated early by an increase in nuclear and total cell size. TSC cultures thus intrinsically contain heterogeneous cell types to various degrees. To study the degree of heterogeneity in cultured TSCs, we first investigated the coexpression of TSC markers using antibodies against CDX2, EOMES, and ELF5 in TSCs cultured on feeder layers of mouse embryonic fibroblasts (MEF) in the presence of MEF-conditioned TSC-medium (MCM), human recombinant FGF4 (hrFGF4), and heparin (F4H) (Figures 2(a)–2(c)). Double-IF staining for EOMES and CDX2 showed a surprising degree of mutual staining. Predominantly, cells at the periphery of colonies showed strong CDX2 staining, while only very weak EOMES signal could be observed. These CDX2 positive cells exhibited a markedly enlarged nucleus in comparison to CDX2 and EOMES double positive cells, indicating that they may have differentiated from TSCs that normally present as cells with small nuclei and cell bodies. Colabelling for EOMES and CDX2 (Figure 1(a)), as well as EOMES and ELF5 (Figure 1(b)), and CDX2 and ELF5 (Figure 1(c)) was very consistently found in small cells, likely representing bona fide TSCs. In larger, more differentiated cells, the CDX2 signal is only partially overlapping with EOMES and ELF5 (Figures 1(a) and 1(c)), while ELF5- and EOMES-staining are predominantly overlapping in all cell types (Figure 1(b)).

To allow for the analysis of TSCs that harbour a stemness-indicating reporter gene, we generated TSCs from E3.5 blastocysts carrying the *Eomes* ^GFP^ knock-in allele [45]. To characterize resulting *Eomes* ^GFP^ TSCs, we compared GFP-reporter expression with *α*-EOMES antibody staining. *Eomes* ^GFP^ reporter and EOMES protein expression widely overlap and highest levels of EOMES protein staining were reflected by strongest reporter activity (Figure 2(d)). As an exception, cells undergoing mitosis loose nuclear EOMES staining, while cytoplasmic GFP expression remains clearly detectable. Highest levels of GFP-reporter expression were found in populations of small cells that morphologically qualify for bona fide TSCs. In summary, our marker analysis in cultured TSCs demonstrates that only the combination of multiple stemness markers allows for unambiguous identification of bona fide TSCs. However, the generation of TSCs carrying the *Eomes* ^GFP^ allele is a suitable tool for the isolation of pure populations of undifferentiated TSCs (GFP^high^) that are characterised by high-level expression of* Eomes*.

### 3.3. Variable Experimental Setups Delineate the Transcriptional Characteristics of Bona Fide TSCs

**Figure 3:**
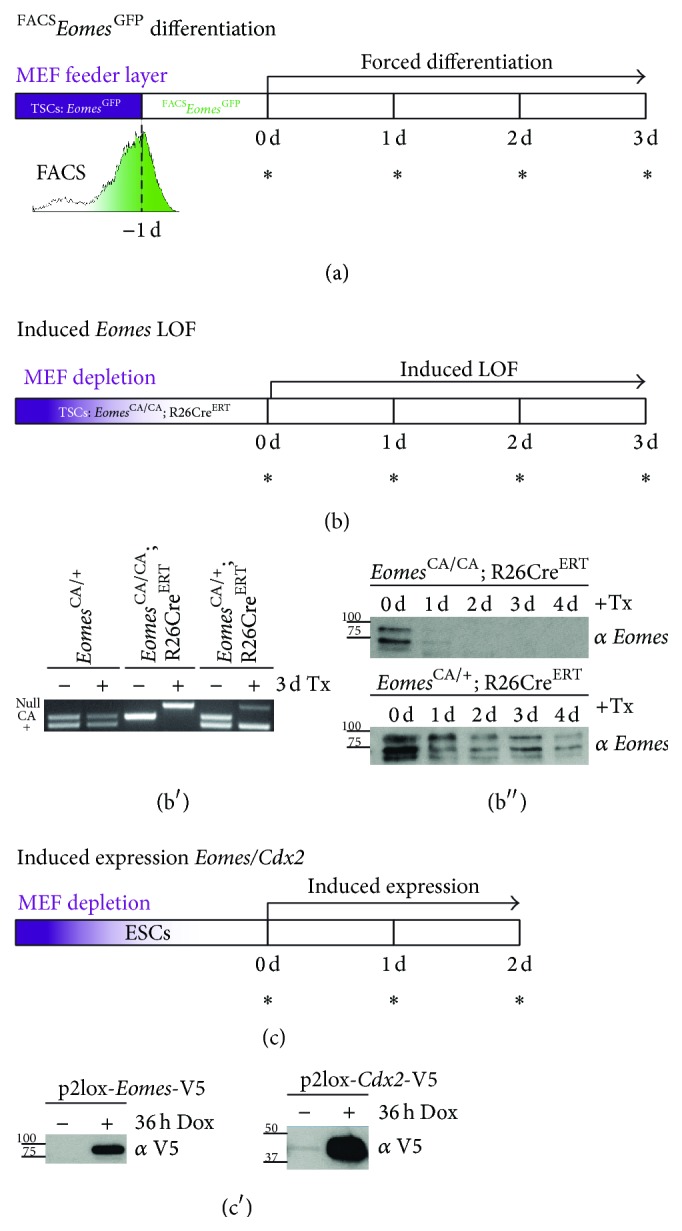
Experimental approaches for the detection and manipulation of the core TSC maintenance factors* Eomes* and* Cdx2*, followed by gene expression profiling. Three complementary experimental approaches were chosen for expression profiling of (a, b) TSCs and (c) mES cells with inducible expression of* Eomes* or* Cdx2*. (a) For the expression profiling of bona fide TSCs and early stages of differentiation, *Eomes* ^GFP^ TSCs were FACS-enriched for GFP^high^ cells and forced towards differentiation by withdrawal of stemness maintaining conditions for 3d. (b) For the identification of* Eomes*-regulated target genes, TSCs harbouring* Eomes* conditional alleles (*Eomes* ^CA^) in combination with a tamoxifen-inducible Cre-estrogen receptor allele (R26Cre^ER^) were used for conditional inactivation* in vitro*. Cells were tamoxifen-treated for 24 h and (b′) Cre-mediated excision was monitored by genotyping PCR, and (b′′) presence of EOMES protein was analysed by Western blot. (c) To transcriptionally profile the initiation of* Eomes*- and* Cdx2*-induced target genes during ES cell to TSC conversion, mES cells with doxycycline-regulated expression were induced for 48 h. (c′) The expression of V5-tagged EOMES or CDX2 protein was monitored by Western blot. MEF, mouse embryonic fibroblast; TSC, trophoblast stem cell; asterisks (**∗**) indicate the different time points of gene expression profiling by gene arrays.

*Eomes* ^GFP^ TSCs allow for the enrichment of GFP^high^ cells representing fully undifferentiated and thus bona fide TSCs. These should serve as a suitable source to delineate the transcriptional signature of TSC stemness.* Eomes* maintains TSCs in an undifferentiated state and the genetic deletion of* Eomes* prohibits TSC maintenance and induces differentiation [34, 41]. In reverse, the expression of* Eomes* or* Cdx2* (and other factors) in mES cells initiates the transcriptional programme that induces lineage conversion towards the TSC lineage [3]. To define the transcriptional signature of TSCs in their bona fide stemness state downstream of* Eomes* and* Cdx2*, we thus employed three experimental settings followed by genomewide expression analyses (Figure 3). First, we enriched *Eomes* ^GFP^ TSCs for GFP^high^ cells and compared cells under stemness maintaining culture conditions with cells that were differentiated by withdrawal of MCM, F4H, and MEFs by gene expression profiling at daily intervals over a period of three days (Figure 3(a)). In the second approach, we induced Cre-mediated gene deletion in TSCs that homozygously carry an* Eomes* conditional allele in combination with a tamoxifen- (Tx-) inducible Cre-estrogen receptor fusion (*Eomes* ^CA/CA^; R26Cre^ERT^) [47] and monitored gene expression at 24 h intervals for three days (Figure 3(b)). The recombination efficiency was monitored by PCR (Figure 3(b′)) and EOMES levels by Western blot, showing that EOMES protein was almost absent for 24 h after Tx-administration (Figure 3(b′′)). Third, to identify genes that are positively regulated by* Eomes* or* Cdx2*, we generated mES cells with inducible overexpression of both transcription factors. mES cells with inducible expression were generated by site-specific introduction of cDNAs including an N-terminal fusion to a V5-tag [48] into the engineered doxycycline-inducible* Hprt* locus of p2lox mES cells using inducible cassette exchange (ICE) [49]. Resulting mES cells allow for temporally regulated and moderate overexpression levels of* Eomes* or* Cdx2* in mES cells as monitored by Western blot (Figures 3(c) and 3(c′)). Expression profiles of mES cells were performed before doxycycline induction and at 24 and 48 h following induced expression to identify the early induced genes downstream of* Eomes* or* Cdx2* that initiate lineage conversion of mES cells towards the TE lineage. All three experimental settings were subsequently used for gene array based transcriptional profiling (see Supplementary Table S3).

### 3.4. Comparative Expression Profiling Reveals the Transcriptional Signature of TSCs

**Figure 4:**
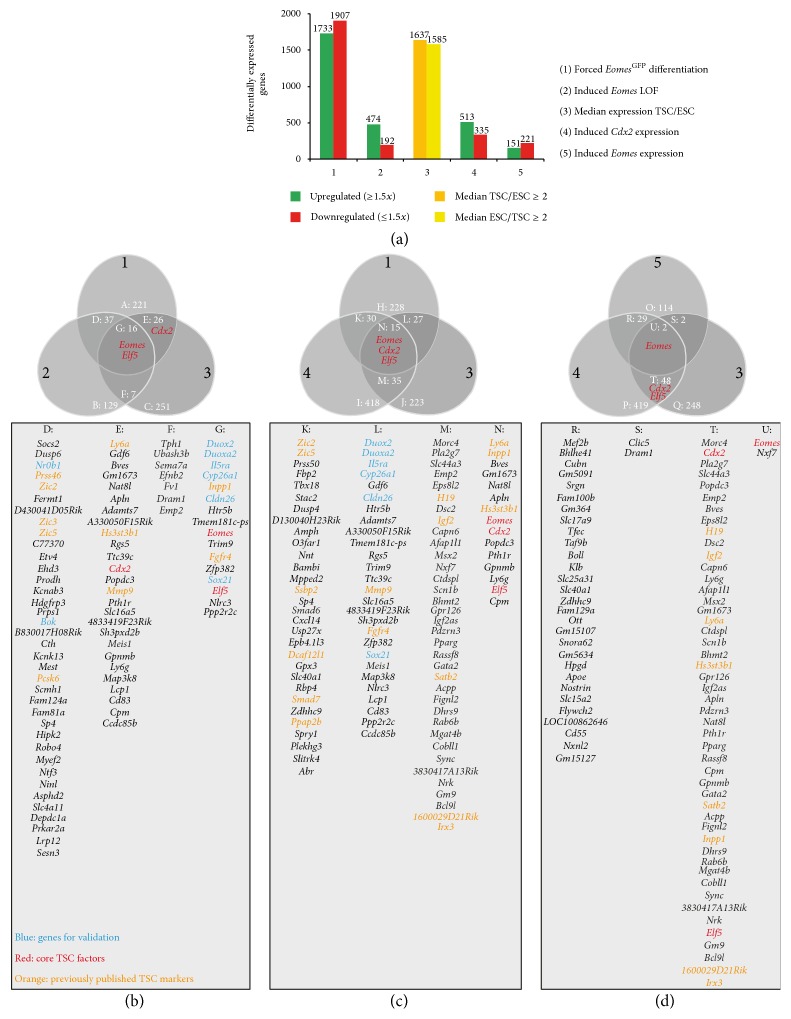
Comparative gene expression analysis reveals candidate TSC marker genes. (a) Genome-wide expression analysis generated five data sets of differentially expressed genes resulting from different experimental settings as shown in Figure 3. (1) The first group contains regulated genes after 3 days of differentiation of *Eomes* ^GFP-high^ FACS-enriched TSCs when compared to undifferentiated *Eomes* ^GFP-high^ TSCs. (2) The second group represents differentially regulated genes 3 days following* Eomes* gene deletion in TSCs (LOF, loss-of-function). (3) The third group comprises genes which show significantly increased or decreased median expression in TSCs versus mES cells, (4, 5) and the fourth and fifth group are differentially expressed genes 2 days after induced expression of* Cdx2* or* Eomes*, respectively. The bar chart indicates numbers of differentially expressed genes for each gene group. (b–d) Venn diagrams indicate groups of intersecting, differentially expressed genes. The genes of resulting groups are listed in the table below each Venn diagram. (b) Comparison between the 300 most strongly downregulated genes in differentiating *Eomes* ^GFP^ FACS-enriched TSCs, the 189 genes downregulated following* Eomes* deletion, and the 300 genes with highest expression ratio between TSCs and mES cells. Of note,* Cdx2*,* Eomes*, and* Elf5* expression levels decrease during TSC differentiation. The loss of* Eomes* function does not significantly reduce* Cdx2* expression after 3d. In (c) the set of differentially regulated genes after* Eomes* gene deletion was exchanged with those 498 genes that were upregulated following* Cdx2*-induction in mES cells. In (d) genes that were induced by* Eomes* or* Cdx2* expression in mES cells were compared to 300 genes with the highest expression ratio in TSCs versus mES cells. Note that* Eomes* is neither significantly upregulating* Elf5* nor* Cdx2* expression. Differences in number of genes listed in (a) and in (b–d) result from genes with multiple transcripts but identical gene symbols. The selected cut-off criteria for genes to be included in datasets were a positive or negative fold change ≥ 1.5 in response to treatment and a *p* value ≤ 0.05. Genes selected for further analyses are highlighted in blue, core genes of the TSC positive feedback loop in red, and genes that were previously identified as TSC markers in orange.

To reveal the transcriptional signature that constitutes and/or maintains a stable TSC state downstream of the key transcription factors* Eomes* and* Cdx2*, we performed a combined analysis of our independent gene array datasets and identified those genes that showed differential expression in multiple experiments (Figure 4 and Supplementary Table S4). All experimental interventions resulted in gross changes of differential gene expression that were defined by changes in expression above 1.5-fold with a *p* value below 0.05 in three biological replicates and a mean level of gene expression above a set background threshold. To identify genes that showed differential expression in multiple experiments, we analysed the intersections of genes that were among (1) the 300 most downregulated genes during differentiation of FACS-enriched Eomes^GFP-high^ TSCs, (2) the 189 genes that were downregulated following induced* Eomes* deletion, (3) the 300 genes that showed the highest relative expression in TSCs compared to mES cells as assessed by the ratio of median expression values in TSCs to the median expression values in mES cells, (4) the 498 genes that were upregulated in response to induced* Cdx2* expression, and (5) the 147 genes that were upregulated in response to induced* Eomes* expression in mES cells (Figure 4 and Supplementary Table S4). The intersections of differentially expressed genes are represented in Venn diagrams and corresponding groups of genes are listed in tables (Figures 4(b)–4(d) and Supplementary Table S4).

In a first comparative analysis, we investigated the intersection of those genes that were (1) downregulated in differentiating *Eomes* ^GFP-high^ TSCs, (2) downregulated in TSCs following induced deletion of* Eomes*, and (3) presented with a high relative expression in TSCs in comparison to mES cells (Figure 4(b)). 16 genes (group G) matched these criteria and thus were considered candidate* Eomes* target genes, which are lost during differentiation of TSCs. Within this group were the known specific TSC markers,* Elf5,* and* Eomes* itself. Additionally, this group contained novel genes that were not previously described in TSCs, such as* Duox2* and* Duoxa2* or* Cldn26. Cdx2* was not included in the intersection, since the deletion of* Eomes* caused only a mild reduction of* Cdx2* within the short time interval of the experiment.

Next, we compared genes that were (1) downregulated during differentiation of *Eomes* ^GFP-high^ TSCs, that were (4) positively regulated by* Cdx2* in mES cells, and that were (3) TSC-specific (Figure 4(c)). The resulting group of 15 genes included* Eomes*,* Elf5*, and* Cdx2* itself.

To compare the early transcriptional changes induced by* Cdx2* or* Eomes* that initiate the conversion of mES cells to TSC-like cells, we analysed the intersecting TSC-specific genes (3) that were upregulated following two days of* Cdx2* (4) or* Eomes* (5) induction (Figure 4(d)). Surprisingly, except for* Eomes*, none of the known TSC factors was significantly upregulated by both* Cdx2* and* Eomes* after two days of induced expression. However, this might reflect the previous observations that* Eomes* is not as effective during the conversion of mES cells to TSCs in comparison to* Cdx2*. While* Cdx2* expression initiates TSC-specific gene expression within 48 hours, including upregulation of* Eomes* and* Elf5, Eomes* expression fails to effectively induce TSC-specific genes within the 2-day interval, despite positive regulation of published* Eomes* targets, such as* Fgf5, Mesp2,* and* Mixl1* [50, 51].

**Figure 5:**
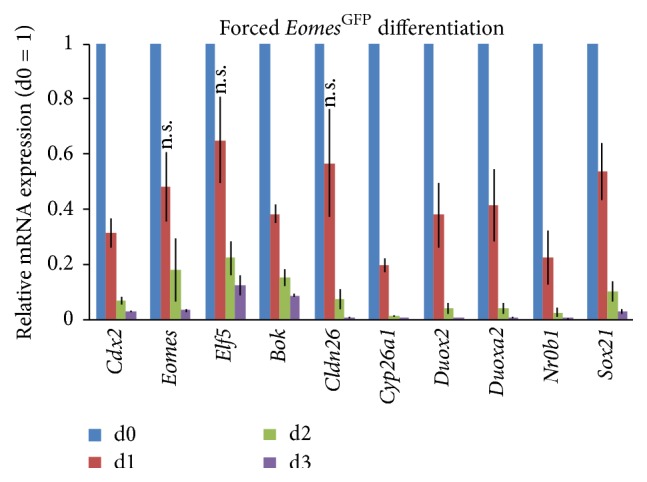
Genes identified by expression arrays and comparative analyses are downregulated during TSC differentiation when validated by qPCR. mRNA expression levels for genes selected by dataset comparisons were quantified at 24 h intervals by qRT-PCR of undifferentiated FACS-enriched *Eomes* ^GFP-high^ TSCs at day 0 (d0) and after forced differentiation for three days (d1–d3). Expression levels are depicted as means of biological triplicates relative to d0, which was set to 1. All genes were significantly (*p* value ≤ 0.05) downregulated at different time points unless indicated otherwise (n.s., not significant). Error bars indicate the standard error of the mean. *p* values were calculated according to two-sided Student's *t*-test. All marker genes show grossly reduced expression during the course of 3 days of differentiation.

To validate the gene array data of differentially expressed genes, we performed quantitative RT-PCRs for established TSC marker genes and selected genes that were at intersections of differentially regulated genes in more than one experiment. To validate specific expression in bona fide TSCs, we compared expression levels in FACS-enriched *Eomes* ^GFP-high^ TSCs with levels during forced differentiation by removal of stemness maintaining conditions (Figure 5). All tested known and novel TSC marker genes showed highest expression in undifferentiated TSCs and significantly reduced levels following induction of differentiation with changes in expression over a minimum of two magnitudes (Figure 5).

### 3.5. Novel Marker Genes Label TSCs during Development of the Early Placenta

**Figure 6:**
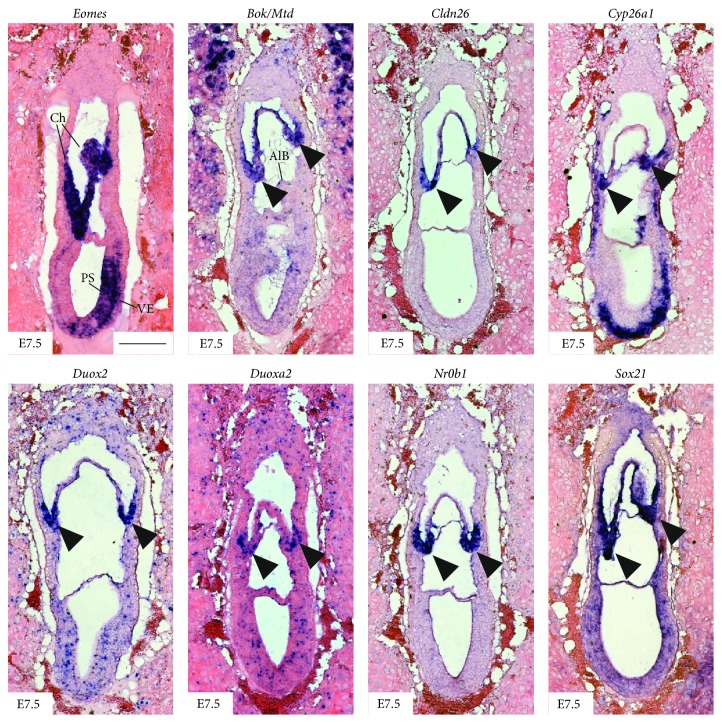
Differentially expressed genes show regional specific expression in the chorion of E7.5 embryos.* In situ* hybridisation analysis on sagittal sections of E7.5 mouse embryos reveals specific expression of* Eomes* as positive control and novel TSC marker genes* Bok/Mtd*,* Cldn26*,* Cyp26a1, Duox2*,* Duoxa2*,* Nr0b1*, and* Sox21*, in the entire chorion, or limited expression in the chorionic hinge (indicated by arrowheads). AlB, allantoic bud; Ch, chorion; PS, primitive streak; VE, visceral endoderm. Scale bar: 200 *μ*M.

To generally validate if the approach revealed novel markers for TSCs during embryonic development, candidate genes were further analysed by* in situ* hybridisation using histological sections of E7.5 embryos. We focused on genes with previously unknown functions in the murine TE, such as* Bok/Mtd*,* Duox2*,* Duoxa2*,* Nr0b1*, and* Sox21*. To our knowledge, specific surface markers for TSCs that would allow for antibody-mediated FACS-sorting have not been identified so far. Thus, we additionally analysed expression of the transmembrane protein-coding gene* Cldn26* (*Tmem114*). For* Sox21*, it was shown that it is involved in the regulation of intestinal and pluripotent stem cells and is induced by* Sox2*, an important stem cell factor in both mES cells and TSCs [52, 53]. Accordingly,* Sox21* was also included into the following analysis. Expression of* Cyp26a1* in the trophectoderm was previously described and together with* Eomes* served as a positive control for expression in the chorion. Of the seven tested novel marker genes, six showed expression in entire chorion (*Bok*,* Cldn26*, and* Sox21*) or were more specifically restricted to the chorionic hinge (*Nr0b1*,* Duox2*, and* Duoxa2*) (Figure 6).

In conclusion, the* in situ* hybridisation analysis revealed that the multimodal expression profiling of TSCs in culture serves as a valuable resource for the identification of novel TSC marker genes* in vitro*, but also for TSCs of the developing placenta.

## 4. Discussion

In the present report we describe a resource for the transcriptional signature of TSCs. We used genetically modified TSCs and mES cells to experimentally interfere with stemness maintaining regulation of TSCs downstream of* Eomes* and* Cdx2*, followed by the assessment of transcriptional changes. The combined analysis of expression data resulted in a comprehensive list of candidate TSC marker genes, of which a handful were tested and positively validated for their specific expression in cultured undifferentiated TSCs and in the chorion of the gastrulating embryo. Thus, the presented expression data will serve as a valuable resource for further studies of stemness maintaining regulatory circuitries of TSCs.

Using TSCs that harbour the *Eomes* ^GFP^ reporter allele allowed for the purification of GFP^high^ TSCs that by morphology and in their transcriptional signature likely resemble bona fide TSCs without contaminating early differentiating TSCs that are normally found in TSC cultures. Corresponding expression data of GFP^high^ TSCs thus resemble the actual signature of bona fide TSCs, underscored by the rapid, gross, and early changes in gene expression that we found during their differentiation. Expression profiles of TSCs in stemness maintaining conditions and during induced differentiation, either by removal of MCM and F4H or by genetic interference with the TSC regulatory circuitry, were previously reported and had revealed partially overlapping gene lists [53–55]. However, the validation of candidates from our comparative expression analysis identified several additional and novel TSC marker genes. Among those is the transcription factor* Sox21* that is strongly expressed in the chorion of the E7.5 embryo and is downregulated during differentiation of TSCs in culture. Interestingly, it was previously demonstrated that* Sox21* is induced by* Sox2*, and, unlike* Sox2* [53],* Sox21* negatively regulates transcription of* Cdx2* in mES and colon cancer cells [52]. This obvious difference in expression in mES cells and TSCs makes* Sox21* an interesting candidate that, like* Sox2* [53], might act differently in the circuitry of TSC and mES stemness factors. Functions of* Sox21* and other novel candidates in TSC biology and TE development will be addressed in ongoing studies.

The importance of* Cdx2* as lineage determining transcription factor was apparently demonstrated by the ability to convert mES cells to TSCs when overexpressed in mES cells [3], even though more recent studies suggest that this lineage conversion might not be complete at the phenotypic, transcriptional, and epigenetic level [56].* Eomes* similarly has the capacity to induce TSC fate; however,* Eomes* seems less potent and possibly induces even less complete lineage conversion [3]. This notion is underscored by our datasets, which display more profound transcriptional responses following* Cdx2* in comparison to* Eomes *expression, when induced from the identical doxycycline-inducible locus. This difference might, at least partially, arise from the reduced induction of* Elf5* in* Eomes*-expressing mES cells which is known as a central component of a positive feed-forward regulation of the core TSC circuitry [25].

While both CDX2 and EOMES were previously used as markers for TSCs, coimmunofluorescence staining of cultured TSCs revealed a remarkable heterogeneity of labelling. Only a subset of TSCs that can be morphologically distinguished by a small cell and nucleus size consistently showed colabelling of CDX2 and EOMES, together with ELF5. We suspect that these cells resemble bona fide TSCs. It will require further fate analysis to reveal if the presence of individual key TSC factors primes future fate decisions, similar to lineage specifying roles of key pluripotency factors during embryonic development and in differentiating mES cells [50, 57]. It is noteworthy that EOMES is also detected in parietal trophoblast giant cells (pTGC) (Figure 1(e)) and thus it is tempting to speculate that differentiation towards the trophoblast giant cell lineage might be promoted by EOMES.

The immune-detection of EOMES, CDX2, and ELF5 in the chorion at late gastrulation stages revealed the presence of TSCs until E9.0–E9.5 in the remaining chorion, and the absence of EOMES-positive cells at later stages. These findings are in line with studies by Uy et al. that demonstrated the presence of TSCs that can be isolated and cultured until the 11-somite stage but not afterwards [31]. Another report that used an Eomes::GFP BAC-transgenic reporter [32] detected GFP positive cells in the margins of the E14.5 placenta. However, the nature of these cells was not defined in detail. We were unable to detect similar cells using EOMES-antibody staining. Thus, we conclude that TSCs are indeed only maintained until E9.5 in the remaining portions of the chorion from which they can be isolated as previously reported [31].

In summary, this study contributes to the characterisation of TSCs during early phases of placentogenesis and of TSCs in culture. We demonstrate that TSCs exhibit a remarkable degree of heterogeneity with respect to protein levels of key TSC transcription factors. TSCs are lost around E9.5 from their stem cell niche in the chorion. We used complementary genetic tools of cultured TSCs and ES cells to determine the transcriptional profile of TSCs in their fully undifferentiated and early differentiating state. Comparative analysis revealed several new TSC marker genes. Presented data can be used as a valuable resource for future studies of TSCs and the corresponding transcriptional regulatory network.

## Supplementary Material

Supplementary Table S1: List of antibodies used for immunofluorescence and western blotting at indicated dilutions.
Supplementary Table S2: List of primers used for qPCR and to generate in situ riboprobes. Restriction sites attached to the primers to ensure directed cloning of the PCR product are underlined.
Supplementary Table S3: Gene expression array data sets.
Supplementary Table S4: Gene groups of Venn diagram intersections.
