# Supplementary material for: A Resource for the Transcriptional Signature of Bona Fide Trophoblast Stem Cells and Analysis of Their Embryonic Persistence
Source: Stem Cells Int. 2015 Dec 14;2015:218518. doi: 10.1155/2015/218518 (PMC4691490; doi:10.1155/2015/218518)
Supplement: Supplementary file 1 — Supplementary Table S1: List of antibodies used for immunofluorescence and western blotting at indicated dilutions. Supplementary Table S2: List of primers used for qPCR and to generate in situ riboprobes. Restriction sites attached to the primers to ensure directed cloning of the PCR product are underlined. Supplementary Table S3: Gene expression array data sets. Supplementary Table S4: Gene groups of Venn diagram intersections. [file 218518.f1.zip › 218518.f1/Supp. mat.docx]

**SUPPLEMENTARY INFORMATION**

**Supplementary Table S1. Antibodies used for immunofluorescence**

| **antigen** | **conjugated** | **isotype** | **supplier** | **Cat. #** | **dilution** |
| --- | --- | --- | --- | --- | --- |
| CDX2 |  | mouse IgG1, kappa | BioGenex | AM392 | 1:50 |
| ELF5 |  | goat IgG | Santa Cruz | sc-9645 | 1:250 |
| EOMES |  | rabbit IgG | Abcam | Ab23345 | 1:1000 |
| V5 |  | mouse IgG2a | Serotec | MCA1360 | 1:1000 |
| mouse IgG (H+L) | Alexa Fluor 647 | goat IgG | Invitrogen | A-21236 | 1:500 |
| goat IgG (H+L) | Alexa Fluor 647 | donkey IgG | Invitrogen | A-21447 | 1:500 |
| rabbit IgG (H+L) | Alexa Fluor 647 | goat IgG | Invitrogen | A21245 | 1:500 |
| rabbit IgG (H+L) | Cy3 | donkey IgG | Jackson Labs | 711-165-152 | 1/1000 |
| mouse IgG (H+L) | Cy3 | donkey IgG | Jackson Labs | 715-165-150 | 1/1000 |

**Supplementary Table S2: Primers used for quantitative RT-PCR or for *in situ* riboprobe generation (restriction sites for cloning are underlined)**

| **primer name** | **name** | **sequence** | **direction** | **purpose** | **Restriction enzyme** |
| --- | --- | --- | --- | --- | --- |
| GK15020113 | Bok/Mtd | AGGTAGTGTCCCTGTATTCCG | FW | qPCR |  |
| GK15020114 | *Bok/Mtd* | AAGGTCTTGCGTACAAACTCC | RV | qPCR |  |
| GK15020115 | *Cldn26* | ATCGGCACCGACTTCTGGTA | FW | qPCR |  |
| GK15020116 | *Cldn26* | GCTGACCGTCACATTCTCCT | RV | qPCR |  |
| GK15020117 | *Cyp26a1* | AAGCTCTGGGACCTGTACTGT | FW | qPCR |  |
| GK15020118 | *Cyp26a1* | CTCCGCTGAAGCACCATCT | RV | qPCR |  |
| GK15020119 | *Duox2* | AAGTTCAAGCAGTACAAGCGAT | FW | qPCR |  |
| GK15020120 | *Duox2* | TAGGCACGGTCTGCAAACAG | RV | qPCR |  |
| GK15020121 | *Duoxa2* | GACGGGGTGCTACCCTTTTAC | FW | qPCR |  |
| GK15020122 | *Duoxa2* | CCCACGGATTCCAGGCAAG | RV | qPCR |  |
| GK15020129 | *Nr0b1* | CCAACACGACGCAGGAAATG | FW | qPCR |  |
| GK15020130 | *Nr0b1* | TGGTGTCAATGTTCAGACTCCA | RV | qPCR |  |
| GK15020131 | *Nr0b1* | CGGATGATGCAGAGAGAGTACC | FW | qPCR |  |
| GK15020132 | *Nr0b1* | AATGATGGGCCTGAAAAAGAGTT | RV | qPCR |  |
| GK15020143 | *Sox21* | CACAACTCGGAGATCAGCAA | FW | qPCR |  |
| GK15020144 | *Sox21* | TGTAGTCGGGATGCTCCTTC | RV | qPCR |  |
| GK15020145 | *Sox21* | GTCTCATCCTTCCTCCCTCC | FW | qPCR |  |
| GK15020146 | *Sox21* | CAGAGTAAGGCTGCCCAGAG | RV | qPCR |  |
| qPCR Eomes for | *Eomes* | GACCTCCAGGGACAATCTGA | FW | qPCR |  |
| qPCR Eomes rev | *Eomes* | GGCCTACCAAAACACGGATA | RV | qPCR |  |
| GK14010091 | *Cldn26* | AAACTAGTTCGGGACTCTGGAGAACTTG | FW | *in situ* | SpeI |
| GK14010092 | *Cldn26* | AACTCGAGAGGTGATAAGGGCTCCACAC | RV | *in situ* | XhoI |
| GK14010099 | *Duox2* | AAACTAGTAGAATGTGGAGAAGGCCTGC | FW | *in situ* | SpeI |
| GK14010100 | *Duox2* | AACTCGAGAAAGGGAGCTGAGGGGTTTG | RV | *in situ* | XhoI |
| GK14010101 | *Duoxa2* | AAACTAGTGTCCTCACGCCTTACTACGG | FW | *in situ* | SpeI |
| GK14010102 | *Duoxa2* | AACTCGAGCGAATGAACGCCAAAATCCTAATC | RV | *in situ* | XhoI |
| GK14010103 | *Cyp26a1* | AAACTAGTGAACATTCGCGCCAAGATCC | FW | *in situ* | SpeI |
| GK14010104 | *Cyp26a1* | AACTCGAGCCACGGGACTGTAGTAGAGAC | RV | *in situ* | XhoI |
| GK14030107 | *Bok/Mtd* | AAACTAGTCATGGGGCAAGGTAGTGTCC | FW | *in situ* | SpeI |
| GK14030108 | *Bok/Mtd* | AACTCGAGCTGGCTTCTCCTGCATTAGC | RV | *in situ* | XhoI |
| GK14030109 | *Sox21* | AAACTAGTCAGAGATCTCGTCGTCGTCG | FW | *in situ* | SpeI |
| GK14030110 | *Sox21* | AACTCGAGGAAGGATGAGACGCAAAAAG | RV | *in situ* | XhoI |
| GK14030111 | *Cldn26* | AAACTAGTGCACCGACTTCTGGTACATC | FW | *in situ* | SpeI |
| GK14030112 | *Cldn26* | AACTCGAGCTGGCTTTCTGGCTTCTGTC | RV | *in situ* | XhoI |
| mw_41 | *Nr0b1* | AAAACTAGTAGGGTGGCATCCTCTACTCT | FW | *in situ* | SpeI |
| mw_42 | *Nr0b1* | AAACTCGAGTCAATGTTCAGACTCCAGCA | RV | *in situ* | XhoI |

**Supplementary Table S3. Gene expression array data sets**

“ANOVA Results” show genes sorted according to their fold change after three days of forced differentiation of FACS-enriched *Eome*s^GFP^ TSCs starting with most downregulated genes in row 2. Additional gene-specific information and gene symbols are provided in columns A-D, median expression values in TSCs, mES cells or TSC- relative to mES cell-based subsets of data are provided in columns E-G. Fold changes and p-values resulting from the individual experimental setups are provided in columns H-AU, more specifically for days one to three of differentiation of *Eome*s^GFP^ TSCs relative to undifferentiated TSCs at day 0 in columns H-M; for one to three days followinginduced *Eomes*-deletion compared to day 0 in columns N-S and relative to the *Eomes*^CA/+^ control at corresponding days in columns T-AA; for up to 2 days of induced *Cdx2* or *Eomes* overexpression in mES cells compared to uninduced day 0 in columns AB-AE and AL-AO, respectively; and for *Cdx2* or *Eomes* overexpressing mES cells compared to control mES cells at corresponding time points in columns AF-AK or AJ-AU, respectively.

**Supplementary Table S4: Gene groups of Venn diagram intersections**

The groups of genes shown in the Venn diagrams of Figure 4 are referred to as “Venn diagram intersections”. These gene lists contain the genes corresponding to the intersections between datasets in Figure 4 B-D. The gene groups A-U correspond to Venn diagram sectors A-U in Figure 4B-D.The genes are sorted according to their fold changes in each group beginning with highest changes in row 3.
